# Supplementary material for: A crowdsourced analysis to identify ab initio molecular signatures predictive of susceptibility to viral infection
Source: Nat Commun. 2018 Oct 24;9:4418. doi: 10.1038/s41467-018-06735-8 (PMC6200745; doi:10.1038/s41467-018-06735-8)
Supplement: Supplementary file 1 — Supplementary Information File [file 41467_2018_6735_MOESM1_ESM.pdf]

Supplementary Materials for

A crowdsourced analysis to identify *ab initio* molecular signatures predictive of susceptibility to viral infection

Fourati et al.

Supplementary Figures

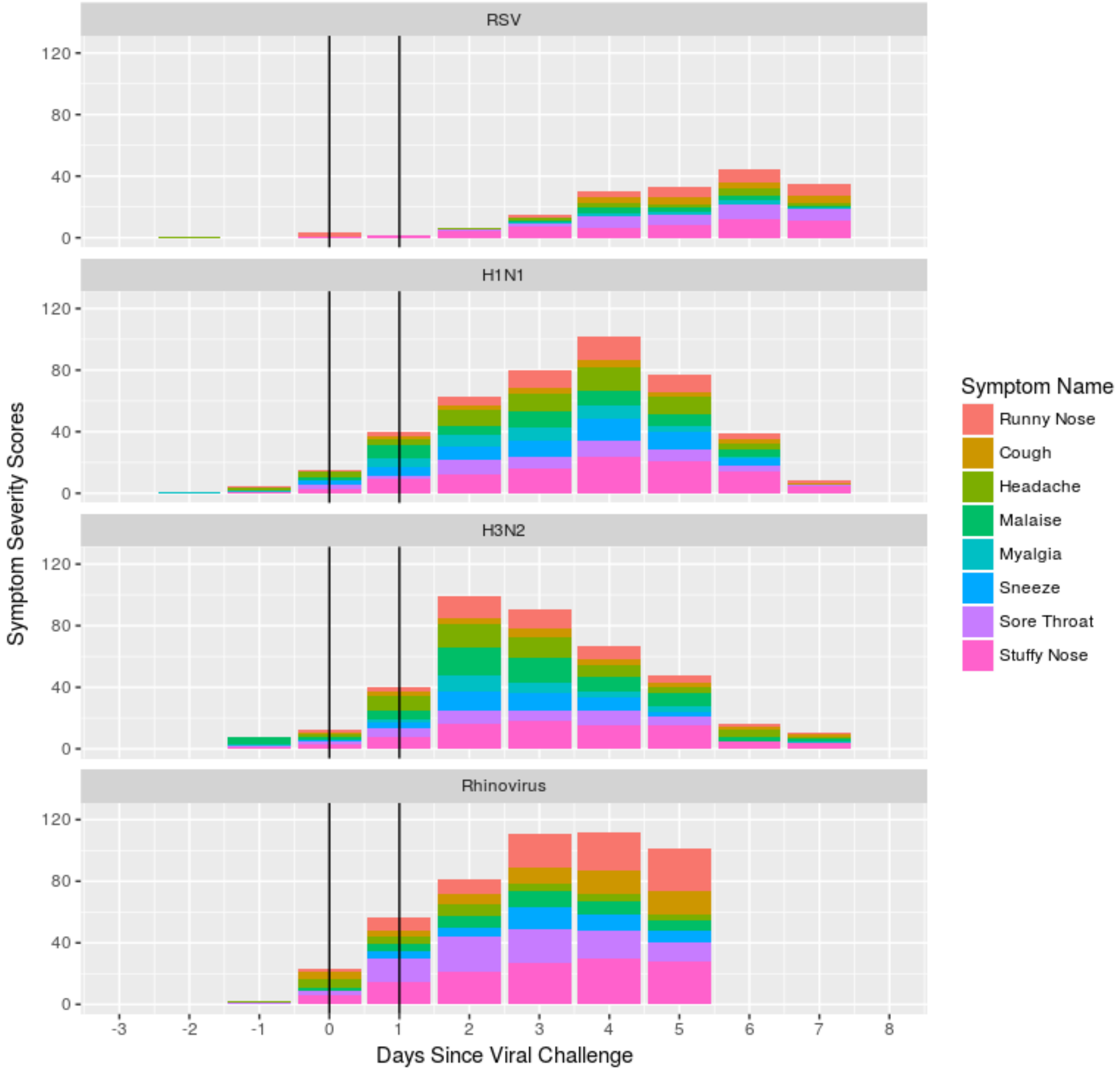

Supplementary Figure 1. Total aggregated symptom load by virus (RSV, H1N1, H3N2, Rhinovirus). While self-reported symptom distributions differ across the different viruses, in each case peak symptoms occur at least one day after the latest time point examined in this study (24 hours post-viral exposure).

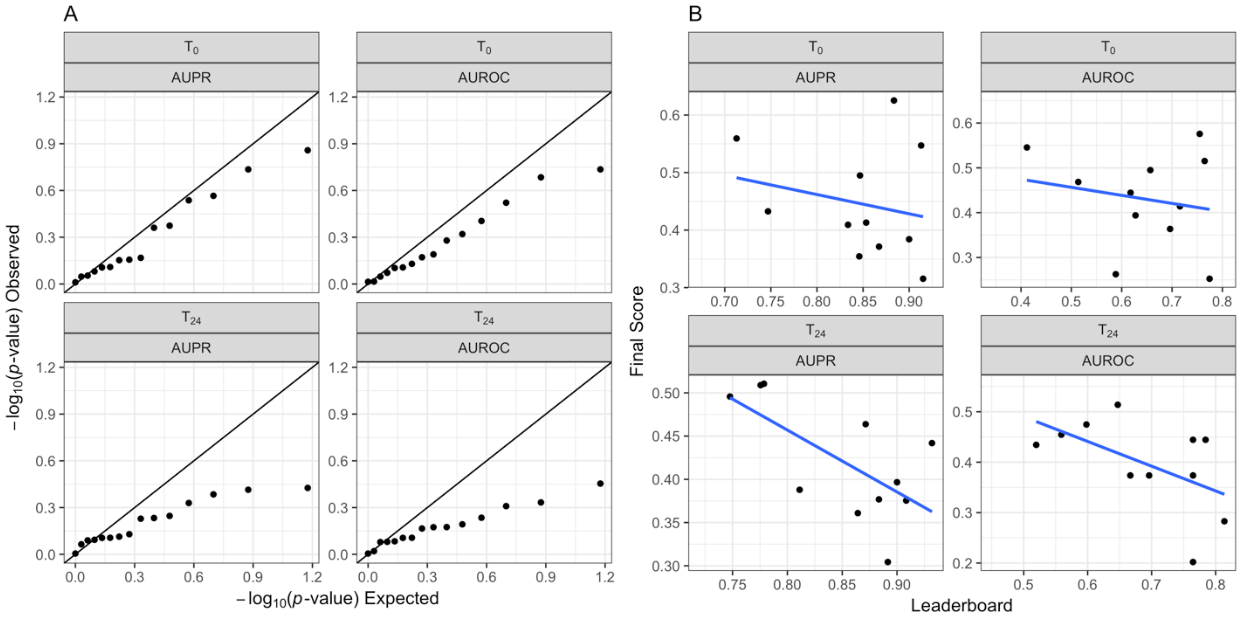

Supplementary Figure 2. **Models show inability to predict viral shedding** (**A**) Observed -$\log_{10}$(p-value) versus the null expectation for submitted predictions for classifying viral shedding (SC1) demonstrates a lack of enrichment (Kolmogorov–Smirnov test for enrichment p-values 0.94, 0.95, 0.82 and 0.95, for AUPR(T_0), AUROC(T_0), AUPR(T_{24}) and AUROC(T_{24}), respectively). (**B**) Correlations between scores from the leaderboard test set and independent test set for SC1 are negative (r = -0.22, -0.19, -0.65, and -0.54 for AUPR(T_0), AUROC(T_0), AUPR(T_{24}) and AUROC(T_{24}), respectively), suggesting overfitting of the training and leaderboard data.

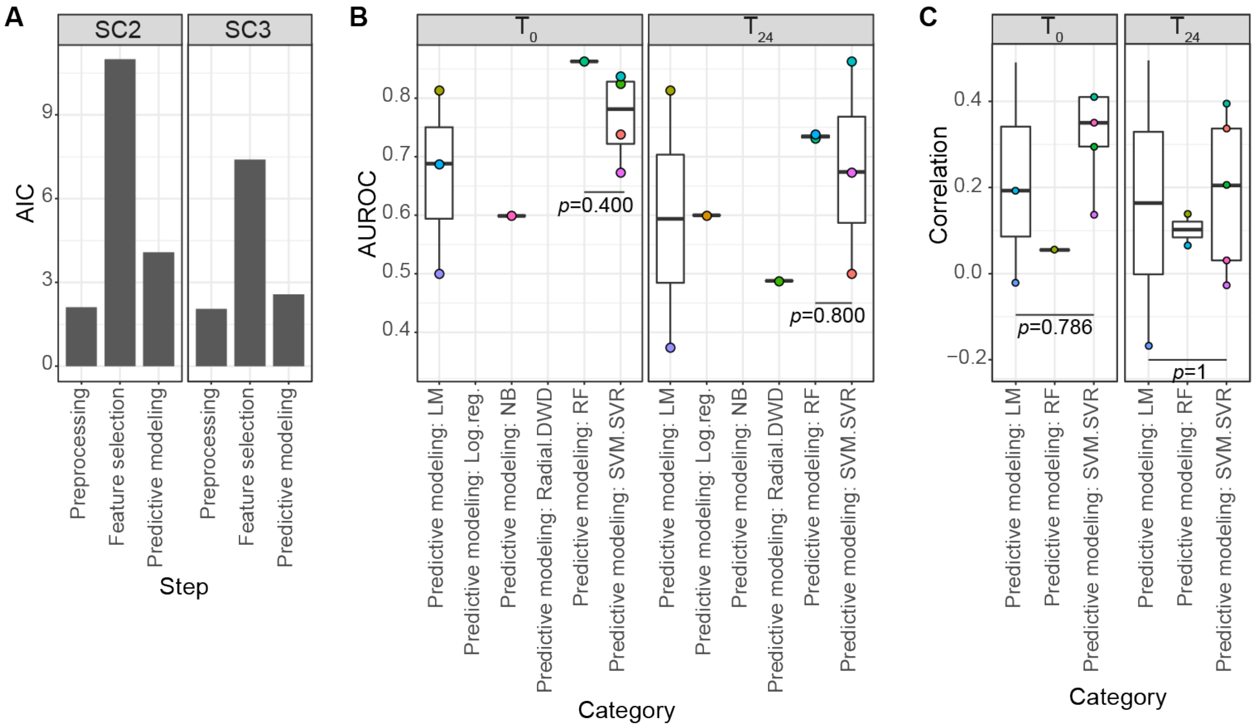

Supplementary Figure 3. Preprocessing and predictive modeling approaches leading to better predictive ability. (**A**) Akaike information criterion (AIC), an estimate of the relative information loss under a predictive model (the smaller the AIC values the better is the trade-off between the goodness of fit and the simplicity of a model) for models representing each of the three major steps in predictive model building. Analysis was performed separately for SC2 and SC3. (**B**) Area under a ROC curve (AUROC) as function of predictive modeling method used to build predictive models of presence of symptoms (SC2). A Wilcoxon rank-sum test was used to assess the variation of prediction ability across the methods. LM: linear least square regression model; Log. reg.: logistic regression; NB: naive bayes; Radial DWD: Radial distance Weighted Discrimination; RF: random forest; SVM: support vector machine; SVR: support vector regression. (**C**) Pearson correlation (Correlation) as function of predictive modeling approaches used to build predictive models of symptoms severity (SC3). A Wilcoxon rank-sum test was used to assess the variation of prediction ability across the methods. On the boxplots (**B-C**), the lower whisker, the lower hinge, the mid hinge, the upper hinge and the upper whisker correspond to $-1.5\times$IQR from the 1st quartile, the 1st quartile, the median, the 3rd quartile and $1.5\times$IQR from the 3rd quartile of the AUROC/Pearson Correlation, respectively.

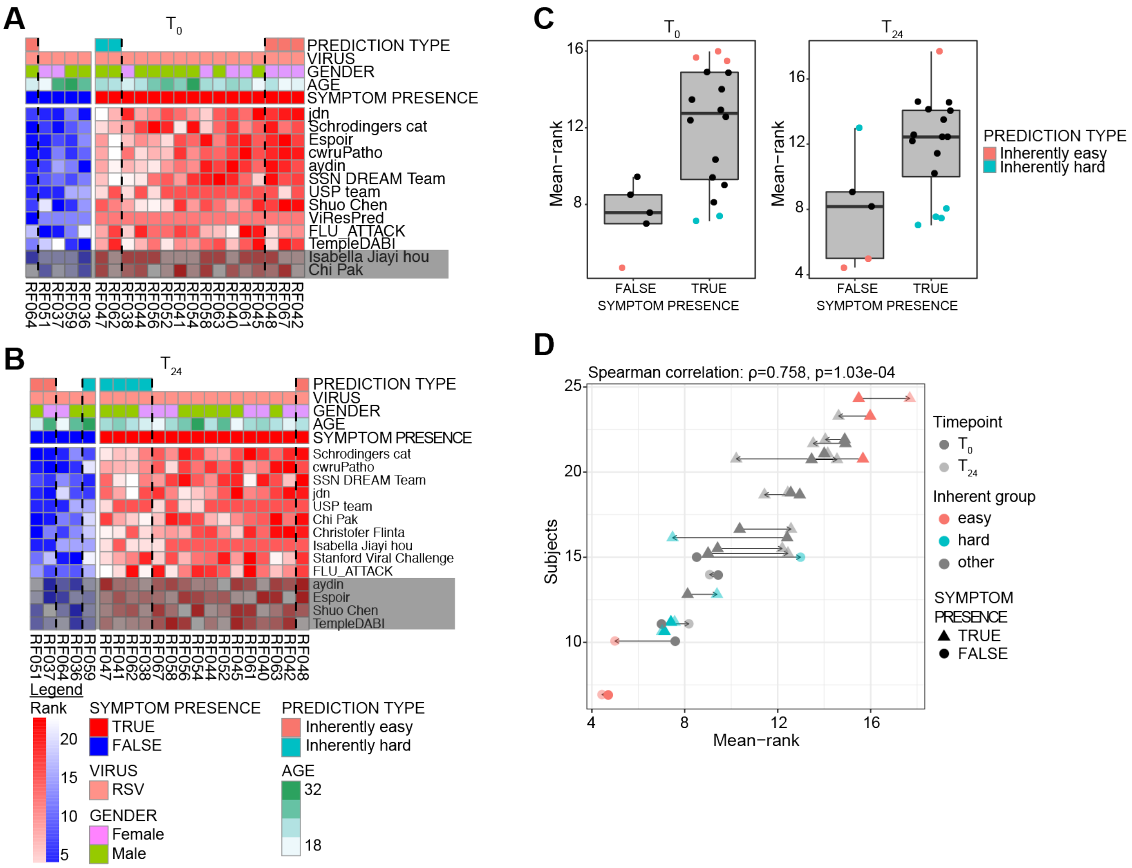

Supplementary Figure 4. **Subjects inherently difficult to predict both at T_0 and T_{24}.** Heatmaps of the predictions of the symptomatic score classifiers for the Independent test set. Predictions were either binary outcome (for 3 teams at T_0 and 2 teams at T_{24}) or continuous probability (for 10 teams at T_0 and 12 teams at T_{24}). Predictions were transformed to ranks in order to be comparable across teams. (**A**) T_0 subjects and (**B**) T_{24} subjects were ordered left to right from the subject predicted by the teams as asymptomatic (*i.e.* symptom presence = FALSE) to the subject predicted by the teams as symptomatic (*i.e.* symptom presence = TRUE). Teams with AUROC < 0.5 (highlighted in grey) were not used for the ordering of the subjects. (**C**) Prediction type groups were identified by investigating the distribution of the mean-rank. Inherently hard subjects that presented symptoms were defined as having mean-rank below the median of the mean-rank of all asymptomatic subjects. Similarly, inherently hard subjects that did not presented symptoms had mean-rank above the median of symptomatic subjects. Inherently easy asymptomatic subjects were defined by having mean-rank strictly below the 1st quartile of asymptomatic subjects while inherently easy symptomatic subjects had mean-rank had mean-rank strictly above the 3rd quartile of the mean-ranks of symptomatic subjects. On the boxplot, the lower whisker, the lower hinge, the mid hinge, the upper hinge and the upper whisker correspond to $-1.5\times$ IQR from the 1st quartile, the 1st quartile, the median, the 3rd quartile and $1.5\times$ IQR from the 3rd quartile of the mean-rank respectively. (**D**) Scatter plot of the average prediction by the teams (x-axis) for each subject (y-axis) by timepoint. Lines connect subjects from the same subjects. Subjects are colored by their prediction type group. Spearman's correlation coefficient and *t*-test were used to evaluate the correlation between T_0 predictions and T_{24} predictions.

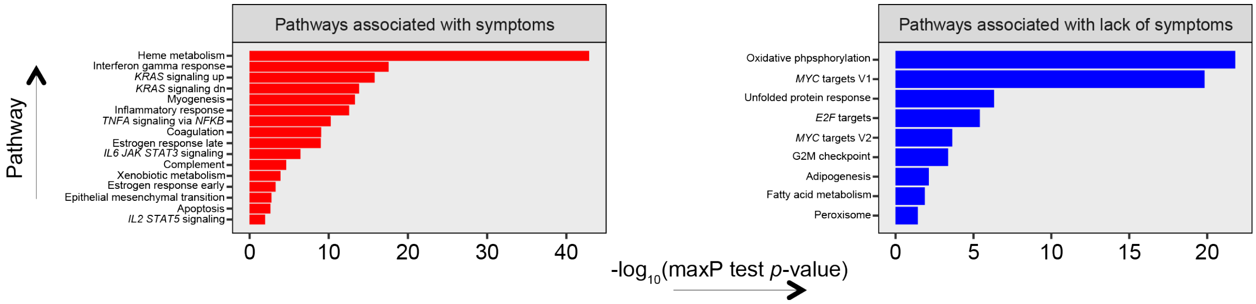

Supplementary Figure 5. Heme metabolism best predicts symptoms across time points and subchallenges. Pathways associated with symptoms and lack of symptoms across time points (T_0 and T_{24}) and subchallenges (SC2 and SC3). The pathways that were enriched at each timepoint for each subchallenge at an adjusted *p*-value < 0.05 was considered. The statistical significance of each pathway was calculated across time points and subchallenges using the maxP test statistic. The x-axis represents the -$\log_{10}$(maxP test *p*-value) value and the y-axis corresponds to the pathways associated with symptoms (in red) and pathways associated with lack of symptoms (in blue) ordered by the decreasing value of -$\log_{10}$(maxP test *p*-value).

Supplementary Tables

Supplementary Table 1. Teams participating in the Challenge

Team	Primary Affiliation(s)	Leaderboard Submission	Provided additional information*	Final Submission
Aganita	Aganitha Cognitive Solutions	x	x	
aydin	Abdullah Gul University	x	x	x
Benjamin Wooden	Icahn School of Medicine at Mount Sinai	x	x	
BulletAnt		x		
CGATeam	Igenomix SL; Fundacion Progreso y Salud; Centre de Regulacio Genomica (CRG)	x	x	
Chengzhe Tian	University of Copenhagen	x	x	
Christofer Flinta	Ericsson Research	x	x	x
cwruPatho	Case Western Reserve University	x	x	x
David Peterson		x		
ES.SJ_PREDICTOMIX	Isfahan University of Medical Sciences; Tabriz University of Medical Sciences	x	x	x
Espoir	University of Washington Tacoma	x	x	x
FLU_ATTACK	INRIA, France; ENS, France; Pasteur Institute, France	x	x	x
GustafssonLab-NordlingLab		x	x	
hackvirus	University of Pennsylvania	x	x	
Isabella Jiayi hou	University of California, San Diego	x	x	x
JayHawks-RVDC	University of South Florida; University of Kansas Medical Center; Moffitt Cancer Center	x	x	
jdn	University of Warsaw	x	x	x
Joshua Burkhart	Oregon Health & Science University	x	x	x
Nautilus	Uppsala University; Polish Academy of Sciences	x	x	
Neo Naoned	Laboratory of Digital Sciences, Nantes; Institut de Calcul Intensif; PIMM, ENSAM ParisTech; National Institute of Informatics, Japan	x	x	
PrecisionHunter	The City University of New York; Shandong University of Finance and Economics; Columbia University	x	x	x
R2heric		x		
Rishemjit Kaur		x		
Ryan Chow	Yale School of Medicine	x	x	
SBiE_KAIST	Korea Advanced Institute of Science and Technology	x	x	
Schrodingers cat	University of Turku	x	x	x
Shosty	University of Pittsburgh	x	x	
Shuo Chen	University of Maryland			x
SSN DREAM Team	Gifu University	x	x	x
Stanford Viral Challenge	Stanford University	x	x	x
Sunil Kumar	Swiss Federal Institute of Technology Lausanne (EPFL)	x	x	
TempleDABI	Temple University	x	x	x
TheBabaYaga	Icahn School of Medicine at Mount Sinai	x	x	
Tony Tan		x		
TXsolo	Icahn School of Medicine at Mt Sinai	x	x	x
USP team	The University of the South Pacific; Griffith University	x	x	x
ViResPred	CSIR-Central Scientific Instruments Organisation Chandigarh, India; University of Hawaii Cancer Center; La Jolla Institute for Allergy and Immunology; ICAR-Indian Agricultural Statistics Research Institute	x	x	x

* Write-up, Code, Predictor list, and/or LOOCVs.

Supplementary Table 2. Methods used by the teams for the predictions of viral shedding and symptoms

Category	Step	Description / Criteria	Number of teams reported (n=24)		
			SC1 (%)	SC2 (%)	SC3 (%)
Excluding subjects	Preprocessing	Exclusion of subjects based on some criteria (SHAM, missing values, etc.)	7 (29)	8 (33)	6 (25)
Normalize	Preprocessing	Use of any specific normalization on the data	14 (58)	12 (50)	11 (46)
Averaging / merging	Preprocessing	Merging of multiple features (or time points) together to generate new features	9 (38)	9 (38)	7 (29)
Discretization	Preprocessing	Division of a continuous attribute into n distinct bins where each bin contains N instances	2 (8)	2 (8)	2 (8)
Machine learning method related	Feature selection	Use of any machine learning-based approach to do the feature selection	13 (54)	12 (50)	11 (46)
Variance based	Feature selection	Filtering out a set of features based on their variance	4 (17)	3 (12)	3 (12)
Correlation based	Feature selection	Filtering out a set of features based on correlation	3 (12)	3 (12)	2 (8)
T-test based	Feature selection	Feature selection based on an approach similar to t-test	3 (12)	3 (12)	2 (8)
Range based	Feature selection	Feature selection based on value range (Defining a cut-off etc.)	3 (12)	4 (17)	3 (12)
Number of features	Feature selection	Number of features used in predictive models	2 (8)	2 (8)	2 (8)
Other	Feature selection	Any other feature that is not explained by the terms above, such as DISR, Feature hashing, etc.	3 (12)	3 (12)	2 (8)
LM	Predictive modeling	Linear model of any form (including "Generalized Linear Model")	6 (25)	5 (21)	5 (21)
Log. reg.	Predictive modeling	Logistic regression	1 (4)	1 (4)	0 (0)
RF	Predictive modeling	Random forest	2 (8)	3 (12)	3 (12)
SVM / SVR	Predictive modeling	Support Vector Machine	9 (38)	6 (25)	7 (29)
NB	Predictive modeling	Naive Bayes	0 (0)	1 (4)	0 (0)
Guass. proc. reg.	Predictive modeling	Gaussian process regression	1 (4)	1 (4)	1 (4)
GBT	Predictive modeling	Gradient Boosted Trees	1 (4)	1 (4)	1 (4)
Radial DWD	Predictive modeling	Radial Distance Weighted Discrimination	0 (0)	1 (4)	0 (0)
Novel	Predictive modeling	Methods that are unconventional and are developed by the group that used them, such as ROSETTA, LIFT, ROAD, etc.	3 (12)	2 (8)	2 (8)

Twenty-four of the thirty four teams participating in the Challenge provided writeups describing the method they used to build their predictive models, which were then classified into methodological category for three processing steps: preprocessing, feature selection, and predictive modeling. The numbers (and proportion) of teams that using each methods are indicated in the table, by subchallenge.
