# Supplementary material for: A crowdsourced analysis to identify ab initio molecular signatures predictive of susceptibility to viral infection
Source: Nat Commun. 2018 Oct 24;9:4418. doi: 10.1038/s41467-018-06735-8 (PMC6200745; doi:10.1038/s41467-018-06735-8)
Supplement: Supplementary file 3 — Description of Additional Supplementary Files [file 41467_2018_6735_MOESM3_ESM.pdf]

Description of Additional Supplementary Files

File Name: **Supplementary Data 1**
Description: **Data sets used in the Challenge.** Eight datasets were used in this challenge. Seven were used for training. For 3 of these, a portion of the samples were held out for use as a leaderboard test set. An independent test set was used for final evaluation of the models trained and optimized on the other 7 datasets.

File Name: **Supplementary Data 2**
Description: **Significantly overlapping genes within virus, subchallenge, and timepoint.** Gene lists used as predictors by challenge participants were compared in order to identify genes that significantly overlapped within virus, subchallenge, and timepoint. The statistical significance of predictor overlap among lists was calculated using the multi-set intersection probability implemented in the SuperExactTest R package. Overlapping predictors associated with *p*-values below 0.005 were considered significant. Significant within-virus predictor lists were generated from teams that separated their predictors by virus (Nautilus, aydin, SSN_Dream_Team, Txsolo, cwruPatho, and Aganita). Significant within-subchallenge predictor lists were generated from teams who provided predictors for both T_0 and T_{24} and both SC2 and SC3 (aydin, SSN_Dream_Team, cwruPatho, jhou). Significant within-timepoint predictor lists were generated from teams who provided predictors for either SC2 or SC3 and provided predictors for both T_0 and T_{24} (aydin, SSN_Dream_Team, cwruPatho, Espoir, jdn, jhou, burkhajo).

File Name: **Supplementary Data 3**
Description: **Gene overlap with previous respiratory viral signatures.** We compared our T_0 and T_{24} gene lists to 42 gene lists found in 12 separate literature sources using Fisher's exact test. Twenty-six unique gene sets significantly overlap with our predictor lists (Benjamini-Hochberg adjusted *p*-value ≤ 0.05). All of the 26 gene sets show significant overlap with our T_{24} gene list; 4 also showed significant overlap with our T_0 gene list.
